# The Phage Lytic Proteins from the *Staphylococcus aureus* Bacteriophage vB_SauS-phiIPLA88 Display Multiple Active Catalytic Domains and Do Not Trigger Staphylococcal Resistance

**DOI:** 10.1371/journal.pone.0064671

**Published:** 2013-05-28

**Authors:** Lorena Rodríguez-Rubio, Beatriz Martínez, Ana Rodríguez, David M. Donovan, Friedrich Götz, Pilar García

**Affiliations:** 1 DairySafe Group, Department of Technology and Biotechnology of Dairy Products, Instituto de Productos Lácteos de Asturias-Consejo Superior de Investigaciones Científicas, Villaviciosa, Asturias, Spain; 2 Animal Biosciences and Biotechnology Laboratory, Animal and Natural Resources Institute, Beltsville Agricultural Research Center, Agricultural Research Service, United States Department of Agriculture, Beltsville, Maryland, United States of America; 3 Interfaculty Institute of Microbiology and Infection Medicine, Microbial Genetics, Eberhard-Karls-Universität Tübingen, Tübingen, Germany; Rockefeller University, United States of America

## Abstract

The increase in antibiotic resistance world-wide revitalized the interest in the use of phage lysins to combat pathogenic bacteria. In this work, we analyzed the specific cleavage sites on the staphylococcal peptidoglycan produced by three phage lytic proteins. The investigated cell wall lytic enzymes were the endolysin LysH5 derived from the *S. aureus* bacteriophage vB_SauS-phi-IPLA88 (phi-IPLA88) and two fusion proteins between lysostaphin and the virion-associated peptidoglycan hydrolase HydH5 (HydH5SH3b and HydH5Lyso). We determined that all catalytic domains present in these proteins were active. Additionally, we tested for the emergence of resistant *Staphylococcus aureus* to any of the three phage lytic proteins constructs. Resistant *S. aureus* could not be identified after 10 cycles of bacterial exposure to phage lytic proteins either in liquid or plate cultures. However, a quick increase in lysostaphin resistance (up to 1000-fold in liquid culture) was observed. The lack of resistant development supports the use of phage lytic proteins as future therapeutics to treat staphylococcal infections.

## Introduction

*Staphylococcus aureus* is a dangerous pathogen responsible for a variety of infections ranging from skin abscesses to fatal sepsis, endocarditis, osteomyelitis, septicemia, pneumonia and meningitis [1]. The emergence of multidrug-resistant strains, especially methicillin-resistant *S. aureus* (MRSA) and vancomycin-resistant *S. aureus* (VRSA) in nosocomial infections, is raising serious concerns within the medical community [2], [3]. There is thus an urgent need for novel therapeutic agents directed against this important pathogen.

Recently, bacteriophages and phage-encoded proteins have been shown as a feasible alternative to antibiotics to overcome this problem [4], [5]. In particular, two phage encoded peptidoglycan (PG) hydrolytic activities (endolysins and virion-associated PG hydrolases) showed antimicrobial activity against Gram-positive pathogens [5], [6]. This has boosted the study of these proteins to be used as therapeutic agents, particularly in external applications [7], [8]. Multiple studies demonstrate the control of both streptococcal (pneumonia, endocarditis, otitis media, meningitis) and *Bacillus anthracis* (intraperitoneal) infections in mice by phage lysins. These results support the application of endolysins to treat human and animal infections [7], [8]. Noteworthy are the staphylococcal infections caused by MRSA strains. In this regard, the intraperitoneal administration of the endolysin MV-L from phage phiMR11 protected mice against MRSA septic death [9]. Similar results were obtained with LysGH15 since the intraperitoneal administration of the lytic enzyme 30 min after MRSA infection was sufficient to guarantee survival of the mice for up to 60 days after treatment [10].

Endolysins and PG hydrolases in general, target the cell wall of both Gram-positive and Gram-negative bacteria, where they cleave covalent bonds. *S. aureus* PG is a complex molecule composed of a sugar backbone of alternating *N*-acetylglucosamine and *N*-acetyl muramic acid residues coupled by β (1→4) linkages. The glycan polymer is in turn linked covalently to short stem peptides which are cross-linked to opposite stem peptides via a pentaglycine (Gly5) bridge generating a three-dimensional network that surrounds the staphylococcal cell [11]. In some genera like *Staphylococcus*, endolysins have evolved acquiring multiple catalytic domains, which confer certain substrate specificities [12]. However, for most of endolysins described to date no biochemical data confirming activity on PG for these domains are available.

The main advantage in the use of endolysins as antimicrobials is the non-detection of bacterial resistance after their repetitive use. Bacterial strains resistant to phage endolysins have not been reported to date, despite efforts to find them. Repeated exposure of *Streptococcus pneumoniae* to lysin Pal [13] and *B. anthracis* to lysin PlyG [14] did not result in the development of lysin-resistant bacteria. In *S. pneumoniae*, the absence of resistance was associated with the presence of choline as a cell wall component essential for pneumococcal viability, which acts at the same time as receptor for the lytic enzymes [15].

**Figure 1:**
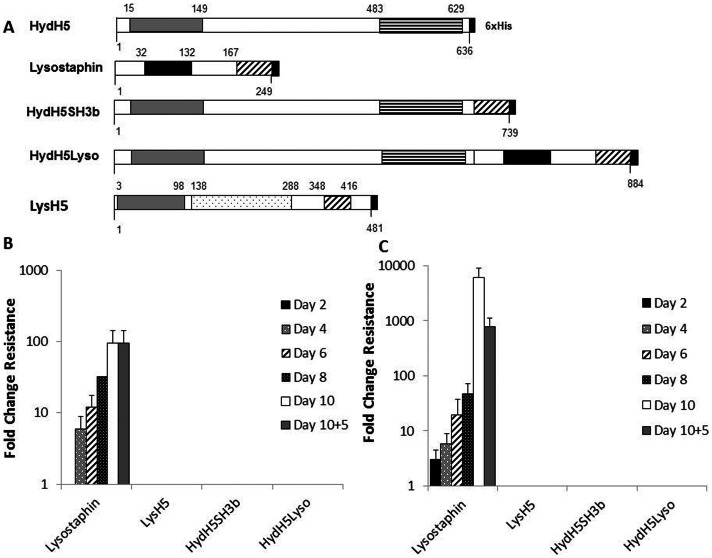
Modular organization and resistance development of lytic enzymes. A) Modular organization of lytic enzymes containing one (lysostaphin), two (LysH5 and HydH5SH3b) or three (HydH5Lyso) catalytic domains. Large black box: endopeptidase domain; Diagonal stripes: SH3b domain; Grey box: CHAP domain; Black dots: amidase-2 domain; Horizontal stripes: LYZ2 domain. Homology: 54% between LysH5 SH3b and Lysostaphin SH3b domains; 30% between LysH5 CHAP and HydH5 CHAP domains. B) Resistance development in solid medium. Plate lysis method using 1∶2 serial dilutions of each protein spotted onto a *S. aureus* Sa9 lawn. C) Resistance development in liquid medium. Minimal Inhibitory Concentration (MIC) repeated exposure assay using 1∶2 serial dilutions of each protein added to 5×10^5^ CFU/well of *S. aureus* Sa9. In both assays, cells surviving at ½ MIC were used as an inoculum for each subsequent round of exposure. The change in susceptibility is presented as fold change from day 1 to 10 of exposure. Error bars are the means ± standard deviations of two independent assays.

The endolysin LysH5 (481 amino acids) encoded by phage vB_SauS-phiIPLA88 (phiIPLA88) was previously characterized and determined by homology to have three putative domains, an N-terminal cysteine, histidine-dependent amidohydrolases/peptidase (CHAP) domain, an amidase-2 domain, and a C-terminal SH3b cell wall-binding (CWB) domain (Fig. 1A). LysH5 is able to inhibit *S. aureus* growth in milk [16] and shows a synergistic antimicrobial effect with the bacteriocin nisin [17].

The phage phiIPLA88 virion-associated PG hydrolase (HydH5) was also characterized [18]. By homology screening, it was shown that HydH5 (634 amino acids) has an N-terminal CHAP lytic domain and a C-terminal LYZ2 (lysozyme subfamily 2) lytic domain. An apparent cell wall binding domain was not identified. By combination between lysostaphin and HydH5 domains, fusion proteins (HydH5SH3b and HydH5Lyso) were obtained (Fig. 1A). These proteins exhibited high lytic activity against bovine and human *S. aureus,* methicillin resistant *S. aureus* N315 and human *Staphylococcus epidermidis* strains [19]. A synergistic interaction between these fusion proteins and phiIPLA88 endolysin LysH5 was also observed [19].

In order to perform an in deep characterization of these phage derived proteins, we studied the catalytic activity of the domains included in LysH5, HydH5SH3b and HydH5Lyso by mass spectrometry analysis of the specific cleavage sites in the *S. aureus* PG. We also evaluated the potential of these phage derived proteins as therapeutic agents through measure of the staphylococcal resistance development after repeated exposure to them.

## Materials and Methods

### Bacterial Strains and Culture Conditions

*S. aureus* Sa9 was used as indicator strain for lytic activity [16] and routinely cultivated either in TSB broth (Tryptic Soy Broth, Difco, Franklin Lakes, NJ) at 37°C with shaking or in TSB plates containing 2% (w/v) bacteriological agar (TSA).

### Protein Expression and Purification

Expression and purification of LysH5, and HydH5SH3b and HydH5Lyso were performed as previously described [17], [19]. Lysostaphin was obtained from Sigma (Sigma, Missouri, USA). Lytic activity quantification of the purified proteins was performed by turbidity reduction assays against live *S. aureus* Sa9 cells as previously described [16], [19].

### Determination of Proteins Cleavage Sites in the PG

Purified PG (2 mg) of *S. aureus* SA113 (CeCo Labs, Tübingen, Germany) was incubated overnight at 37°C with 1 µM of HydH5SH3b or HydH5Lyso in a final volume of 250 µl activity buffer A: HEPES 50 mM, NDSB-201(non detergent sulfobetaine) 0.5 M, CaCl_2_ 0.25 mM, MnCl_2_ 0.25 mM, MgCl_2_ 0.25 mM, TCEP (tris(2-carboxyethyl)phosphine) 1 mM, NaCl 24 mM, KCl 1 mM pH 7.5. Similar digests of PG with LysH5 were performed in phosphate buffered saline (PBS). Samples were boiled 3 min to stop the reaction and centrifuged at 14,000×*g* for 5 min to eliminate the insoluble fraction. Soluble muropeptides obtained in each digestion were separated by reverse-phase HPLC using a Nucleosil 100 column (C18; 125×4.6 mm; 5 µm; Maisch GmbH, Ammerbuch, Germany) and a water/0.1% TFA: 80% acetonitrile/0.1% TFA gradient for 150 min at flow rate of 0.5 ml/min. Peaks were then analysed by Liquid Chromatography-Mass Spectrometry (LC-MS) (Agilent Technologies, Waldbronn, Germany).

### Determination of Bacterial Resistance to Lytic Proteins

Resistant development was tested using repeated exposures in both the plate lysis and the minimal inhibitory concentration (MIC) assays. For the plate lysis assay, 2-fold serial dilutions of the proteins were spotted (10 µl) onto a freshly plated lawn of *S. aureus* Sa9 on TSA plates and grown overnight at 37°C. LysH5 dilutions were made in activity buffer B (CHES 50 mM, PEG 3350 0.06%, CaCl_2_ 0.25 mM, MnCl_2_ 0.25 mM, ZnCl_2_ 0.25 mM, TCEP 1 mM, pH 9), starting at 1 µM. HydH5SH3 and HydH5Lyso dilutions were made in activity buffer A starting at 4 µM and lysostaphin dilutions were made in activity buffer A starting at 1 µM. Cells from spots with a not fully cleared lawn (sub-lethal) were scraped, inoculated in 5 ml of TSB and grown to mid log phase (OD_600nm_ 0.4–0.6) to generate a new lawn for the next round of plating and enzyme exposure. After 10 rounds of exposure, cells were grown for 5 additional overnight cultures on TSA plates in the absence of lytic proteins to allow any putative phenotype-reversion of non-genetically altered strains. Finally, a new plate lysis assay was performed with the cultures resulting from these 5 non-selective grow-outs to re-test the sensitivity of the putative resistant cultures to the lytic proteins.

In the MIC assays, for 10 consecutive days, cells were exposed overnight to 2-fold serial dilutions of LysH5, HydH5SH3b, HydH5Lyso and lysostaphin. MICs were performed as previously described [20] using *S. aureus* Sa9 strain. Proteins concentrations started at 0.25 µM for lysostaphin and LysH5 and 3 µM for HydH5SH3b and HydH5Lyso. In every round, 100 µl of the first well with growth (½ MIC) were inoculated into 5 ml of TSB and grown to mid log phase (DO_600nm_ 0.4–0.6). These cultures were used for the next round of MIC exposure. Cells surviving after round 10 were grown for 5 additional rounds in TSB without lytic proteins to allow phenotype-reversion; then, MICs were performed to measure the sensitivity to the proteins after non-selective grow out.

## Results

### Identification of the PG Bond Cleaved by Phage Lytic Proteins

**Figure 2:**
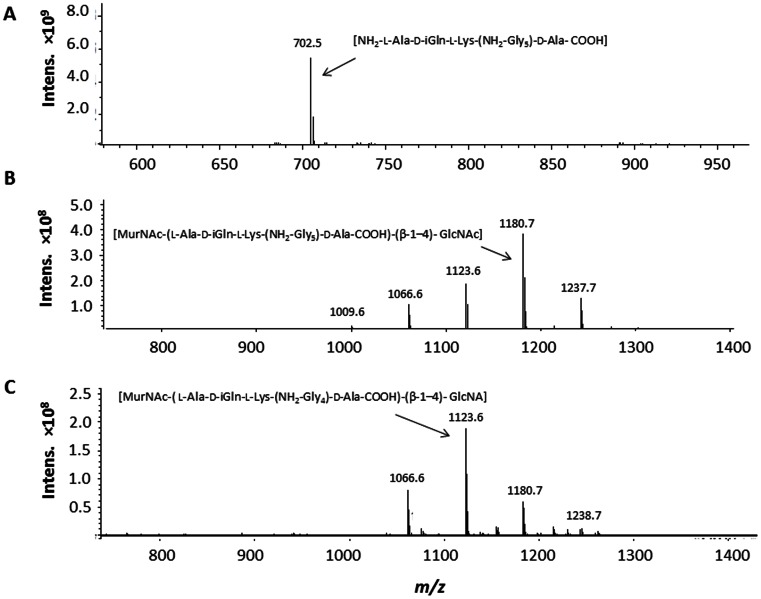
MS analysis, in positive mode, of the overnight digestion of 2 mg of *S. aureus* SA113 peptidoglycan with phage lytic proteins. Peptidoglycan was digested using 1 µM of: A) LysH5, B) HydH5SH3b and C) HydH5Lyso in a final volume of 250 µl buffer HEPES 50 mM, NDSB-201 0.5 M, CaCl_2_ 0.25 mM, MnCl_2_ 0.25 mM, MgCl_2_ 0.25 mM, TCEP 1 mM, NaCl 24 mM, KCl 1 mM pH 7.5 for HydH5SH3b and HydH5Lyso and phosphate buffered saline (PBS) for LysH5. The primary products obtained are indicated.

**Figure 3:**
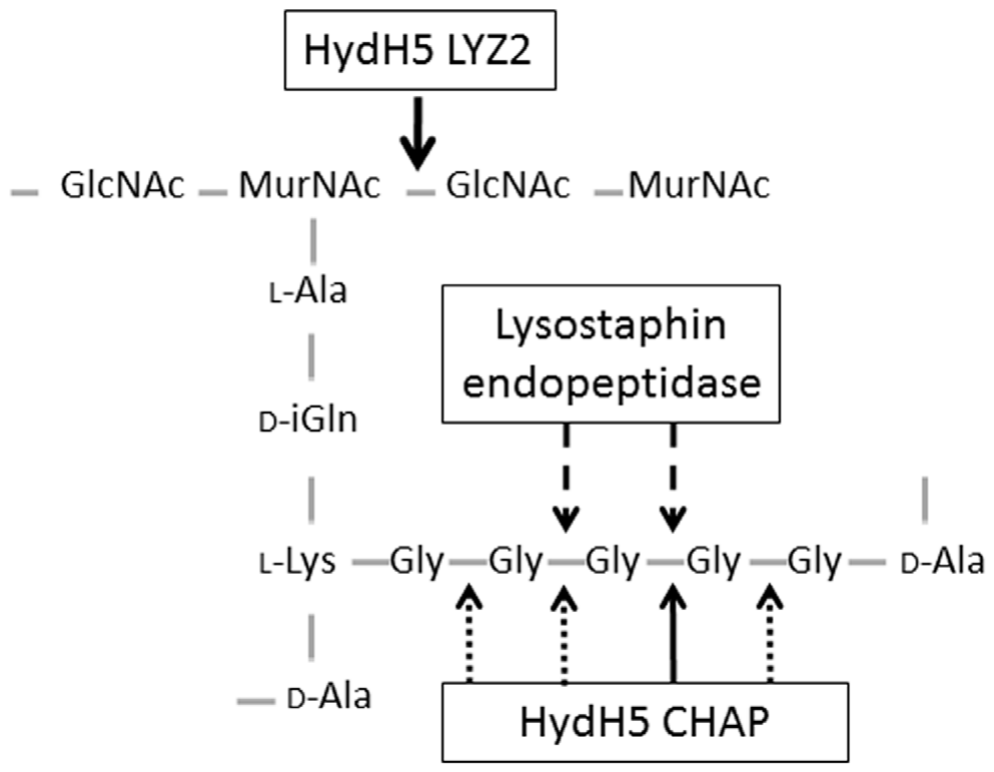
HydH5SH3b and HydH5Lyso cleavage sites on the peptidoglycan structure of *S. aureus*. The glycan chain consists of β- 1,4- linked monomers of N-acetyl glucosamine (GlcNAc) and N-acetyl muramic acid (MurNAc). The stem peptide contains amino acids L-Ala, D-iGln, L-Lys and D-Ala. This peptide is cross-linked to an opposite stem peptide on a separate glycan polymer through a pentaglycine (Gly5) interpeptide bridge. Arrows indicate the bonds targeted by the lytic proteins.

To determine the LysH5, HydH5SH3b and HydH5Lyso PG cut sites, LC-MS was performed on proteins-digested *S. aureus* PG preparations. The LysH5 digestion generated a primary product which in MS yields a peak with *m/z* = 702.5 (Fig. 2A), a measurement that is in close agreement with the structure of the PG cleavage fragment NH_2_-L-Ala-D-iGln-L-Lys-(NH_2_-Gly5)-D-Ala-COOH (calculated MH^+^702.41). To generate this fragment, two enzymatic activities must be present, an amidase that cleaves between the N-acetylmuramic acid and L-alanine of the stem peptide, and an endopeptidase that cleaves either at the termini or within the pentaglycine cross-bridge. Identical MS spectra were obtain for phi11 endolysin (97% homologous to LysH5) which was determined to have both a N-acetylmuramoyl-L-alanine amidase and an endopeptidase activity that cleaves at the termini of the cross-bridge between the last Gly and the D-Ala (Fig. 3) [11]. HydH5SH3b *S. aureus* cell wall digestions gave a predominant peak which yielded an ion in MS with *m/z* 1180.7 (Fig. 2B). The peak ion mass corresponds to the PG fragment with structure MurNAc-(L-Ala-D-iGln-L-Lys-(NH_2_-Gly5)-D-Ala-COOH)-(β-1–4)-GlcNAc. Consistent with the two lytic domains identified in HydH5 via homology screening (Fig. 1A), two enzymatic activities are needed to generate this fragment, a muramidase activity to cleave the sugar backbone, and an endopeptidase activity to cleave the interpeptide cross-bridge. The exact cleavage site of the endopeptidase is not obvious, in so far as a consistent cleavage site anywhere in the pentaglycine bridge would generate a fragment with 5 Gly residues. However, the presence of multiple smaller peaks (*m/z* 1123.6, 1066.6 and 1009.6) that differ in size in one Gly residue, suggests that the endopeptidase does have a preferred cleavage site (largest peak) but might be promiscuous and cleave at multiple Gly-Gly bonds in the interpeptide bridge.

**Figure 4:**
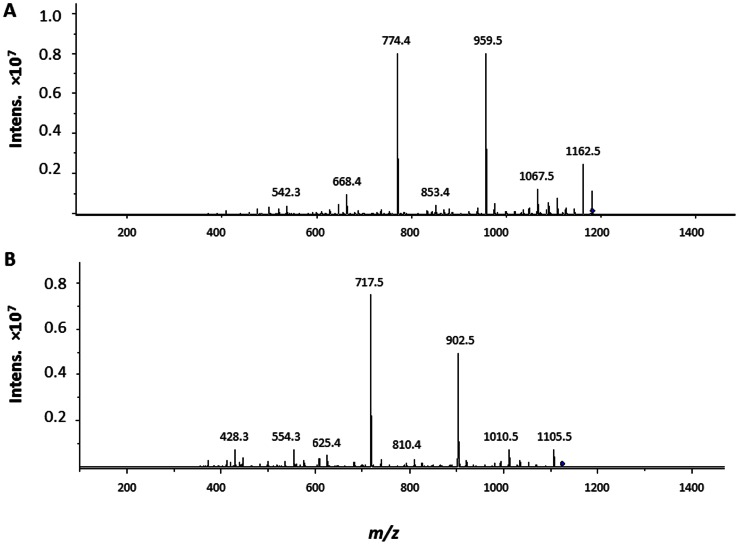
Analysis of the peptidoglycan digestion products obtained with phage lytic proteins. MS/MS analysis, in positive mode, of the main peaks obtained in the *S. aureus* SA113 peptidoglycan digestion with (A) HydH5SH3b and (B) HydH5Lyso. A) MS/MS of the *m/z* = 1180.7 ion yielded two main fragmentation products. *m/z* = 959.5 ion corresponds to the product of removal of the GlcNAc, and *m/z* = 774.5 ion is in agreement with the peptidoglycan structure MurNAc-(L-Ala-D-iGln-L-Lys-(NH2-Gly3). B) MS/MS of the *m/z* = 1123.6 ion yielded two main daughter ions. *m/z* = 717.5 ion corresponds to the peptidoglycan structure MurNAc-(L-Ala-D-iGln-L-Lys-(NH2-Gly2) and *m/z* = 902.5 corresponds to the structure MurNAc-(L-Ala-D-iGln-L-Lys-(NH2-Gly4)-D-Ala-COOH).

PG digestions with the HydH5Lyso fusion (Fig. 2C) yields a peak ion at *m/z* 1123.6 which corresponds closely to a PG fragment consisting of MurNAc- (L-Ala-D-iGln-L-Lys-(NH_2_-Gly4)-D-Ala-COOH)-(β-1–4)-GlcNAc indicating that both the endopeptidase and muramidase domains are active in this construct. However, one smaller peak (*m/z* 1066.6) is also produced. This peak differs in size from the larger peak in one Gly residue. The loss of one Gly residue suggests that the HydH5 endopeptidase is also active in this construct and likely can cleave between Gly residues 1 and 2 or 4 and 5. Further evidence of the cleavage sites comes from MS/MS of the HydH5SH3b PG digestion product *m/z* = 1180.7 ion which yielded two major fragmentation product ions at *m/z* = 774.5 and *m/z = *959.5 (Fig. 4A). The *m/z = *959.5 ion corresponds to the MurNAc- (L-Ala-D-iGln-L-Lys-(NH_2_-Gly5)-D-Ala-COOH)-(β-1–4)- GlcNAc, whereas the compound with the *m/z* = 774.5 is in agreement with the PG structure MurNAc-(L-Ala-D-iGln-L-Lys-(NH_2_-Gly3), suggesting that the remaining 2 Gly residues in the PG fragment are linked to the D-Ala at position four in the stem peptide. This may indicate that the CHAP domain of HydH5SH3b cleaves the bond between Gly residues at positions 3 and 4 of the cross-bridge peptide (Fig. 3). Nevertheless, this could be the preferred cleavage site, as other smaller peaks of the digestion yielded ions with *m/z* 1123.6, 1066.6 and 1009.6 corresponding to similar PG fragments with 4, 3, and 2 Gly residues (Fig. 2B), respectively, and ions with *m/z* 1237.7, 1294.7 and 1351.7 corresponding with 6, 7 and 8 Gly, respectively (data not shown). These data suggest that the HydH5 CHAP domain is a glycyl-glycine endopeptidase that cleaves any Gly-Gly bond of the pentaglycine cross-bridge, with a preferential site between the third and fourth Gly residues. MS/MS of the *m/z* = 1123.6 ion, the main product of the *S. aureus* PG digestion with HydH5Lyso, yielded two main ‘daughter ions’ with *m/z* 717.5 and 902.5 (Fig. 4B). The *m/z* 902.5 ion corresponds to the product of the removal of the GlcNAc in the structure MurNAc- (L-Ala-D-iGln-L-Lys-(NH_2_-Gly4)-D-Ala-COOH)-(β-1–4)- GlcNAc, and *m/z* 717.5 ion corresponds to the PG structure MurNAc-(L-Ala-D-iGln-L-Lys-(NH_2_-Gly2), indicating, as previously, that 2 Gly residues in the MurNAc-(L-Ala-D-iGln-L-Lys-(NH_2_-Gly4)-D-Ala-COOH)-(β-1–4)- GlcNAc ion are likely linked to the terminal D-Ala of the stem peptide. These data suggest that the glycyl-glycine bond between Gly residues 2 and 3 of the interpeptide bridge were likely cut by the lysostaphin endopeptidase domain of HydH5Lyso (Fig. 3), since the preferred cleavage site for the CHAP endopeptidase domain is between Gly 3 and 4 as described above for HydH5SH3b.

### Assessment of *S. aureus* Resistance to Phage Lytic Proteins

In order to assess the ability of *S. aureus* to develop resistance against phage lytic proteins, we initially attempted to select mutants by serial subculture of *S. aureus* Sa9 in the presence of sub-inhibitory concentrations of proteins LysH5, HydH5SH3b, HydH5Lyso. Exposure of *S. aureus* Sa9 to lysostaphin was used as a positive control for resistance development. In two independent subcultures in which the plate lysis method was used, the concentration of lysostaphin that produced a cleared spot increased from 0.03±0.0 µM to 3±1.4 µM at the end of the experiment; a ∼100-fold rise in resistance (Fig. 1B). In contrast, when *S. aureus* Sa9 was exposed to serial dilutions of LysH5, HydH5SH3b and HydH5Lyso, no spontaneous resistant mutants were recovered. Confirmation of this result was obtained by serial subculture in liquid medium in the presence of sub-inhibitory concentrations of these proteins (MIC assay). The lysostaphin MIC increased ∼1000-fold (from 0.003±0.0 µM to 3±1.4 µM) after 10 rounds of exposure (Fig. 1C), whereas serial subculture in liquid medium failed to select resistant variants for LysH5, HydH5SH3b and HydH5Lyso. Noteworthy is the differential stability of lysostaphin resistance in both assays. A remarkable decrease in resistance was observed for isolates derived from liquid culture in the absence of lysostaphin (Fig. 1C) as opposed to a higher stability observed from solid medium-derived mutants (Fig. 1B).

## Discussion

The molecular structure of some phage lytic proteins composed by several catalytic domains seems to be a strategy to guarantee their biological function [12]. We have previously demonstrated that phage lytic proteins from bacteriophage phiIPLA88 are highly effective in the elimination of *S. aureus* strains [16], [18], [19]. In the work presented here, the analysis of PG cut sites for these proteins indicated that all predicted catalytic domains are active. However, the ability to digest purified PG might not reflect the activity on live cells [20]. This is not expected to be the case for HydH5 since the CHAP and LYZ2 domains, as single domains obtained via deletion analysis, have shown similar lytic activity on live cells [18], [19]. This similar activity of both domains contradicts the results previously reported by other authors since one of the two catalytic domains seems to be almost inactive in some modular endolysins [20]–[23]. Similarly, the lysostaphin endopeptidase domain is active in the triple-lytic domain fusion HydH5Lyso, as the specific activity of this construct on live cells is higher than that previously obtained for HydH5SH3b [19].

Our results indicate that CHAP and lysostaphin endopeptidase domains have some overlap in their target bonds. However, we have a conclusive proof of the fully activity of the lysostaphin domain at the C-terminus of the fusion construct which is consistent with the C-terminal position of lysostaphin in a previously reported triple fusion [24]. On the other hand, it was unexpected that the HydH5 CHAP domain would be a glycyl-glycine endopeptidase since other CHAP domains from staphylococcal lysins were reported to be alanyl-glicine endopeptidases [11]. However, mass spectrometry data clearly indicate CHAP domain activity which is supported by the N-terminal position of this domain in the fusion construct (i.e. the same as the native HydH5 protein). Moreover, it is interesting to speculate about the activity of this domain against the classical lysostaphin resistant mutants. Furthermore, the presence of an active LYZ2 domain in the chimeric proteins ensures that this fusion protein will confer antimicrobial activity despite any alteration of the pentaglycine interpeptide bridge.

Development of bacterial resistance is a major issue that should be addressed before implementation of new antimicrobials. We have determined that resistance to the phage lytic proteins LysH5, HydH5SH3b and HydH5Lyso seems to be a rare event as deduced from the absence of *S. aureus* spontaneous mutants able to survive after repeated exposures to the proteins. These results are in concordance with previous studies about endolysins resistance development in which no bacteria resistant to streptococcal phage endolysins were found despite several attempts to find them [25]. It has been hypothesized that phage lytic enzymes have evolved to target essential bonds in the cell wall, making resistance to these enzymes unlikely [26]. Furthermore, the high-affinity binding of some endolysins is directed towards species- or strain-specific cell-wall carbohydrates that are often essential for viability. This is the case of amidases PlyG whose specificity for specific bacilli is achieved by selective binding to a uniquely galactosylated core structure [27], and Pal which contains a choline binding module that attaches the enzyme to choline residues present in pneumococcal envelope [28]. Having so conserved catalytic and binding targets could be a reason for the lack of bacterial resistance development against these phage proteins [13], [14].

On the other hand, bacterial resistance is less likely in response to dual acting lysins compared to single lytic domains lysins [26] due to the need to modify multiple target cleavage sites. Similarly, three active domains are likely to further prevent resistance development [24]. Phage lytic proteins LysH5, HydH5SH3b and HydH5Lyso are endowed of multiple catalytic domains active against both highly conserved (LysH5 amidase domain, HydH5 lysozyme domain) and less conserved bonds (LysH5 CHAP domain, HydH5 CHAP domain). Thus, HydH5 CHAP domain cleaves the pentaglycine bridge which in the case of *S. aureus* is easily modified, as shown in mutants that avoid the action of lysostaphin [29]. Maybe that modification could also hamper HydH5 CHAP activity; however, the presence of an additional active muramidase domain (LYZ2) in HydH5 can ensure the antimicrobial activity of the protein. In this regard, we could not state to what extent the lack of resistance for these phage lytic proteins was due to the presence of multiple catalytic domains or to the addition of activities against high conserved bonds, or both.

### Conclusions

Phage lytic proteins have a potential use as antistaphylococcal agents for treatment of infections caused by *S. aureus* antibiotic-resistant strains and as biocontrol agents in the food industry. Therefore, prior to their use we proved the activity of all their catalytic domains and the lack of resistance development after repeated exposure of *S. aureus* to sub-inhibitory concentrations of LysH5, HydH5SH3b and HydH5Lyso.

## References

[1] Lowy FD (1998) *Staphylococcus aureus* infections. N Engl J Med 339: 520–532. 9709046 10.1056/NEJM199808203390806

[2] Appelbaum PC (2006) MRSA–the tip of the iceberg. Clin Microbiol Infect Suppl 2: 3–10. Review. 10.1111/j.1469-0691.2006.01402.x 16524422

[3] Appelbaum PC (2006) The emergence of vancomycin-intermediate and vancomycin-resistant *Staphylococcus aureus.* Clin Microbiol Infect Suppl 1: 16–23. Review. 10.1111/j.1469-0691.2006.01344.x 16445720

[4] Nelson DC, Schmelcher M, Rodriguez-Rubio L, Klumpp J, Pritchard DG, et al (2012) Endolysins as antimicrobials. Adv Virus Res 83: 299–365. 22748813 10.1016/B978-0-12-394438-2.00007-4

[5] Rodríguez-Rubio L, Martínez B, Donovan DM, Rodríguez A, García P (2012) Bacteriophage virion-associated peptidoglycan hydrolases: potential new enzybiotics. Crit Rev Microbiol doi:10.3109/1040841X.2012.723675. 10.3109/1040841X.2012.723675 22991936

[6] Fenton M, Ross P, McAuliffe O, O’Mahony J, Coffey A (2010) Recombinant bacteriophage lysins as antibacterials. Bioeng Bugs 1: 9–16. 21327123 10.4161/bbug.1.1.9818 PMC3035150

[7] Shen Y, Mitchell MS, Donovan DM, Nelson DC (2012) Phage-based Enzybiotics. In: Hyman P, Abedon ST, editors. Bacteriophages in Health and Disease. Wallingford, UK: CAB International. 217–239.

[8] Schmelcher M, Donovan DM, Loessner MJ (2012) Bacteriophage endolysins as novel antimicrobials. Future Microbiol 7(10): 1–25. 23030422 10.2217/fmb.12.97 PMC3563964

[9] Rashel M, Uchiyama J, Ujihara T, Uehara Y, Kuramoto S, et al (2007) Efficient elimination of multidrug-resistant *Staphylococcus aureus* by cloned lysin derived from bacteriophage phi MR11. J Infect Dis 196: 1237–1247. 17955443 10.1086/521305

[10] Gu J, Xu W, Lei L, Huang J, Feng X, et al (2011) LysGH15, a novel bacteriophage lysin, protects a murine bacteremia model efficiently against lethal methicillin-resistant *Staphylococcus aureus* infection. J Clin Microbiol 49: 111–117. 21048011 10.1128/JCM.01144-10 PMC3020447

[11] Navarre WW, Ton-That H, Faull KF, Schneewind O (1999) Multiple enzymatic activities of the murein hydrolase from staphylococcal phage phi11. Identification of a D-alanyl-glycine endopeptidase activity. J Biol Chem 274: 15847–15856. 10336488 10.1074/jbc.274.22.15847

[12] Oliveira H, Melo LD, Santos SB, Nóbrega FL, Ferreira EC, et al.. (2013) Molecular aspects and comparative genomics of bacteriophage endolysins. J Virol doi:10.1128/JVI.03277–12. 10.1128/JVI.03277-12 PMC3624390 23408602

[13] Loeffler JM, Nelson D, Fischetti VA (2001) Rapid killing of *Streptococcus pneumoniae* with a bacteriophage cell wall hydrolase. Science 294: 2170–2172. 11739958 10.1126/science.1066869

[14] Schuch R, Nelson D, Fischetti VA (2002) A bacteriolytic agent that detects and kills *Bacillus anthracis.* . Science 418: 884–889. 10.1038/nature01026 12192412

[15] García P, García E, Ronda C, Tomasz A, Lopez R (1983) Inhibition of lysis by antibody against phage-associated lysin and requirement of choline residues in the cell wall for progeny phage release in *Streptococcus pneumoniae* . Curr Microbiol 8: 137–140.

[16] Obeso JM, Martínez B, Rodríguez A, García P (2008) Lytic activity of the recombinant staphylococcal bacteriophage PhiH5 endolysin active against *Staphylococcus aureus* in milk. Int J Food Microbiol 128(2): 212–218. 18809219 10.1016/j.ijfoodmicro.2008.08.010

[17] García P, Martínez B, Rodríguez L, Rodríguez A (2010) Synergy between the phage endolysin LysH5 and nisin to kill *Staphylococcus aureus* in pasteurized milk. Int J Food Microbiol 141(3): 151–155. 20537744 10.1016/j.ijfoodmicro.2010.04.029

[18] Rodríguez L, Martínez B, Zhou Y, Rodríguez A, Donovan DM, et al (2011) Lytic activity of the virion-associated peptidoglycan hydrolase HydH5 of *Staphylococcus aureus* bacteriophage vB_SauS-phiIPLA88. BMC Microbiol 11: 138. 21682850 10.1186/1471-2180-11-138 PMC3150257

[19] Rodríguez-Rubio L, Martínez B, Rodríguez A, Donovan DM, García P (2012) Enhanced staphylolytic activity of the *Staphylococcus aureus* bacteriophage vB_SauS-phiIPLA88 HydH5 virion-associated peptidoglycan hydrolase: fusions, deletions, and synergy with LysH5. Appl Environ Microbiol 78(7): 2241–2248. 22267667 10.1128/AEM.07621-11 PMC3302612

[20] Becker SC, Dong S, Baker JR, Foster-Frey J, Pritchard DG, et al (2009) LysK CHAP endopeptidase domain is required for lysis of live staphylococcal cells. FEMS Microbiol Lett 294(1): 52–60. 19493008 10.1111/j.1574-6968.2009.01541.x

[21] Donovan DM, Foster-Frey J, Dong S, Rousseau GM, Moineau S, et al (2006) The cell lysis activity of the *Streptococcus agalactiae* bacteriophage B30 endolysin relies on the cysteine, histidine-dependent amidohydrolase/peptidase domain. Appl Environ Microbiol 72(7): 5108–5112. 16820517 10.1128/AEM.03065-05 PMC1489305

[22] Horgan M, O’Flynn G, Garry J, Cooney J, Coffey A, et al (2009) Phage lysin LysK can be truncated to its CHAP domain and retain lytic activity against live antibiotic-resistant staphylococci. Appl Environ Microbiol 75(3): 872–874. 19047377 10.1128/AEM.01831-08 PMC2632115

[23] Schmelcher M, Korobova O, Schischkova N, Kiseleva N, Kopylov P, et al (2012) *Staphylococcus haemolyticus* prophage ΦSH2 endolysin relies on cysteine, histidine-dependent amidohydrolases/peptidases activity for lysis ‘from without’. J Biotechnol 162(2–3): 289–298. 23026556 10.1016/j.jbiotec.2012.09.010 PMC4062874

[24] Donovan DM, Becker SC, Don S, Baker JR, Foster-Frey JA, et al (2009) Peptidoglycan hydrolase enzyme fusions for treating multi-drug resistant pathogens. Biotech Int 21: 6–10.

[25] Fischetti VA (2008) Bacteriophage lysins as effective antibacterials. Curr Opin Microbiol 11: 393–400. 18824123 10.1016/j.mib.2008.09.012 PMC2597892

[26] Fischetti VA (2005) Bacteriophage lytic enzymes: novel anti-infectives. Trends Microbiol 13(10): 491–496. 16125935 10.1016/j.tim.2005.08.007

[27] Mo KF, Li X, Li H, Low LY, Quinn CP, et al (2012) Endolysins of *Bacillus anthracis* bacteriophages recognize unique carbohydrate epitopes of vegetative cell wall polysaccharides with high affinity and selectivity. J Am Chem Soc 134(37): 15556–15562. 22935003 10.1021/ja3069962 PMC3489029

[28] García E, García JL, García P, Arrarás A, Sánchez-Puelles JM, et al (1988) Molecular evolution of lytic enzymes of *Streptococcus pneumoniae* and its bacteriophages. Proc Natl Acad Sci U S A 85(3): 914–918. 3422470 10.1073/pnas.85.3.914 PMC279667

[29] Kusuma C, Jadanova A, Chanturiya T, Kokai-Kun JF (2007) Lysostaphin-resistant variants of *Staphylococcus aureus* demonstrate reduced fitness *in vitro* and *in vivo* . Antimicrob Agents Chemother 51(2): 475–482. 17101683 10.1128/AAC.00786-06 PMC1797764

